# PCR amplification of a long rDNA segment with one primer pair in agriculturally important nematodes

**DOI:** 10.21307/jofnem-2019-026

**Published:** 2019-06-03

**Authors:** L. K. Carta, S. Li

**Affiliations:** 1 Mycology and Nematology Genetic Diversity and Biology Laboratory, USDA, ARS, Henry A. Wallace Beltsville Agricultural Research Center, Bldg. 010A, Room 110, Beltsville, MD, 20705-2350

**Keywords:** Nematode, Primers, Ribosomal DNA, Taxonomy, Phylogeny

## Abstract

Ribosomal DNA has been a reliable source of taxonomic and phylogenetic markers due to its high copy number in the genome and stable variation with few polymorphisms due to the homogenizing effect of concerted evolution. Typically specific regions are amplified through polymerase chain reaction (PCR) with multiple primer pairs that generate often incomplete and overlapping regions between adjacent segments of 18S, ITS1, 5.8S, ITS2, and 28S rDNA nucleotide sequences when combined in tandem. To improve the efficiency of this effort, a strategy for generating all these molecular sequences at once through PCR amplification of a large ribosomal 3.3 to 4.2 kb DNA target was developed using primer 18S-CL-F3 paired with D3B or a new alternative 28S PCR primer (28S-CL-R) and other well-positioned and ribosomal-specific sequencing primers (including novel primers 18S-CL-F7, 18S-CL-R6, 18S-CL-R7, 18S-CL-F8, 5.8S-CL-F1, 5.8S-CL-R1, 28S-CL-F1, 28S-CL-R3, 28S-CL-F3, 28S-CL-R1, and 28S-CL-F2). The D1 region between ITS2 and 28S boundaries and the flanking sequence between 18S and ITS1 boundaries were fully revealed in this large nucleotide segment. To demonstrate the value of this strategy, the long rDNA segment was amplified and directly sequenced in 17 agriculturally important nematodes from the Tylenchida, Aphelenchida, and Dorylaimida. The primers and their positions may be employed with traditional Sanger sequencing and with next-generation sequencing reagents and protocols.

Eukaryotic nuclear ribosomal DNA (rDNA) is arranged in tandem repeat arrays in the genome. Each repeat unit consists of one copy of small subunit (SSU) 18S, internal transcribed spacers (ITS1 and ITS2), 5.8S, and large subunit (LSU) 28S rDNA, and is separated by an external transcribed spacer (EST) and an intergenic spacer (IGS) (Hillis and Dixon, 1991). The copy number of the repeats within most eukaryotic genomes is high, which provide large quantities of template DNA for PCR. In *Caenorhabditis* spp., for instance, the rDNA copy number was estimated to be as many as 56 to 323 copies within their genomes (Bik et al., 2013). Second, the rDNA polymorphisms among the repeat units are very low within the genome due to concerted evolution (Liao, 1999). These two features make rDNA particularly well suited for taxonomic identification, phylogenetic analysis, and barcoding for nematodes (Blaxter et al., 1998; Floyd et al., 2002; Holterman et al., 2006; Megen et al., 2009; Rodrigues Da Silva et al., 2010). As a result, there have been more than 300,000 nematode rDNA sequences published in GenBank to date. One of the most important means of determining these rDNA sequences is the amplification of the target rDNA loci by polymerase chain reaction (PCR). Usually 18S, 28S, and ITS are amplified separately with different PCR primer pairs; one or more PCR primer pairs are used to amplify 18S to near full length (Carta and Li, 2018), one pair for ITS1, 5.8S, and ITS2 (Ferris et al., 1993; Vrain, 1993; Joyce et al., 1994), and one pair for the D2D3 segment of 28S (Nunn, 1992). This multiple-pair approach is time-consuming and cost-ineffective for the amplification of this long target of approximately, 3.3 to 4.2 kb in length from 18S, ITS1, 5.8S, ITS2 to the D3 of 28S; furthermore, from a probabilistic point of view, the more primer pairs that are applied to amplify this long target, the lower the success rate of the amplification will be. Therefore, minimizing the number of primer pairs is a key to successfully amplifying this long target. In this short technical note, we have tested the PCR amplification of this 3.3 to 4.2 kb rDNA target with one ribosomal primer pair in 17 agriculturally important nematodes and sequenced the resulting amplicons directly with well-positioned and ribosomal-specific sequencing primers.

## Materials and methods

### DNA extraction

Live J2 from *Heterodera* spp. and *Meloidogyne incognita* and live adult nematodes from other taxa described in Table 1 were selected for this study. Template DNA was prepared in the format of one nematode per tube by using freeze-thaw lysis. Before the extraction, the nematodes were washed twice with molecular biology grade water to remove any micro contaminants attached to their bodies. A clean single nematode was picked and transferred to a 0.2 ml PCR tube containing 24 µl of extraction buffer (10 mM Tris pH 8.2, 2.5 mM MgCl_2_, 50 mM KCl, 0.45% TWEEN 20, and 0.05% gelatin, Williams et al., 1992). The tube was submerged in liquid nitrogen for 10 to 15 sec and then placed at 95°C for 2 min in a C1000 Touch^TM^ thermal cycler (Bio-Rad Laboratories, Hercules, CA). This rapid physical disruption was repeated once. Then the tube was subjected to the third cycle of freezing in liquid nitrogen for 10 to 15 sec and then was slow-thawed at room temperature. The thawed sample was lysed with 1 µl of proteinase K (800 U/ml, Sigma-Aldrich, St. Louis, MO) at 60°C for 60 min, followed by 95°C for 15 min to deactivate the proteinase K. At least three single nematodes from each taxon were picked for the individual DNA extraction. All resulting lysates were stored in a −20°C freezer until needed. The DNA extracts from *Ditylenchus*, *Ecumenicus*, and *Radopholus* were prepared previously by using mechanical lysis (Carta et al., 2010).

**Table 1.** Agriculturally important nematodes tested in this study.

Taxa	Origin and locality	PCR primer pair	Ta (°C)	Sequencing primers
*Aphelenchoides fragariae*	Live specimen 104J12, provided by Dr. Paula Agudelo, Clemson University, Clemson, South Carolina	18S-CL-F3 and 28S-CL-R	50	530F, 530R, 1912R, 18S-CL-F2, 18S-CL-R2, 18S-CL-R5, ITS-CL-F2,28S-CL-F1, D2AR, 28S-CL-F3, 28S-CL-R1 and 28S-CL-R
*Bursaphelenchus* sp.	Live specimen 104J7, isolated from walnut twig beetles, Washington	18S-CL-F3 and 28S-CL-R	57	530F, -530R, 18S-CL-R2, 18S-CL-F7,18S-CL-F2, 18S-CL-R5, 18S-CL-R7, 18S-CL-F8, ITS-CL-F2, rDNA15.8S, 28S-CL-F1, D2AR, 28S-CL-F3, 28S-CL-R1, 28S-CL-F2 and 1006R
*Bursaphelenchus xylophilus*	Live specimen 104H46, isolated from Eastern white pine tree, New Hampshire	18S-CL-F3 and 28S-CL-R	57	530F, -530R, 18S-CL-R2, 18S-CL-F7,18S-CL-F2, 18S-CL-R5, 18S-CL-R7, 18S-CL-F8, ITS-CL-F2, rDNA15.8S, 28S-CL-F1, D2A, D2AR, 28S-CL-F3, 28S-CL-R1, 28S-CL-F2 and 1006R
*Crossonema* sp.	Live specimen 104G38, isolated from the rhizosphere of bamboo, Beltsville, Maryland	18S-CL-F3 and D3B	57	530F, 530R, 1912R, 18S-CL-F2, 18S-CL-R2, 18S-CL-R5, ITS-CL-F2,28S-CL-F1, D2AR, 28S-CL-F3, 28S-CL-R1 and 28S-CL-R
*Ditylenchus dipsaci*	Live specimen 85A5, obtained from French Iris in Wisconsin by USDA-APHIS-PPQ interception	18S-CL-F3 and 28S-CL-R	50	530R, 1912R,18S-CL-R2, 18S-CL-F7,18S-CL-F2, 18S-CL-R5, 18S-CL-R7, 18S-CL-F8, ITS-CL-F2, rDNA15.8S, AR28, V2R, 28S-CL-F1, 28S-CL-F3, D2AR, 28S-CL-R1, and 28S-CL-F2
*Ditylenchus* sp.	Live specimen 85C1, isolated from the rhizosphere of alfalfa, Moab, Utah	18S-CL-F3 and 28S-CL-R	50	530R, 1912R,18S-CL-R2, 18S-CL-F7,18S-CL-F2, 18S-CL-R5, 18S-CL-R7, 18S-CL-F8, ITS-CL-F2, rDNA15.8S, 5.8SF, AB28, V2R, 28S-CL-F1, 28S-CL-F3, 28S-CL-R3, 28S-CL-R1, 28S-CL-F2, 1006R
*Ecumenicus* sp.	Live specimen 85G11, isolated from the rhizosphere of alfalfa, St. George, Utah	18S-CL-F3 and D3B	50	530R, 530F,18S-CL-R2, 18S-CL-F7,18S-CL-F2, 18S-CL-R5, 18S-CL-R6, ITS-CL-F2, rDNA15.8S, 5.8SF, V2R, 28S-CL-F1, D2AR, D2A, 28S-CL-R3, 28S-CL-R1, 28S-CL-F2, 1006R
*Helicotylenchus* sp.	Live specimen 104G36, isolated from the rhizosphere of bamboo, Beltsville, Maryland	18S-CL-F3 and D3B	57	530R, 530F,1912R, 18S-CL-R2, 18S-CL-F7,18S-CL-F2, 18S-CL-R5, 18S-CL-F8, ITS-CL-F2, 5.8SF, V2R, 28S-CL-F1, D2AR, 28S-CL-R1, 28S-CL-F2, 28S-CL-R
*Heterodera glycines*	Live J2 specimen Hg20, from the isolate, NL1-RHp originally was collected in on the east shore of Maryland and raised on soybean (*G. max*, cv. Kent) in sand-filled beakers	18S-CL-F3 and D3B	57	530R, 1912R, 18S-CL-R2, 18S-CL-F7,18S-CL-F2, 18S-CL-R7, 18S-Cl-F8, rDNA15.8S, 5.8SF, AB28, 28S-CL-F1, D2A, 28S-CL-F3, 28S-CL-R1, 28S-CL-F2, 1006R, and 28S-CL-R
*Heterodera orientalis*	Live J2 specimen 104F80, isolated from the cyst in the rhizosphere of *Miscanthus*, Beltsville, Maryland.	18S-CL-F3 and D3B	57	530R, 530F, 1912R, 18S-CL-R2, ,18S-CL-F2, 18S-CL-R5, ITS-CL-F2, 28S-CL-F1, D2AR, 28S-CL-F3, 28S-CL-R1, 28S-CL-F2, 28S-CL-R
*Hoplolaimus* sp.	Live specimen 104G35, isolated from the rhizosphere of bamboo, Beltsville, Maryland	18S-CL-F3 and D3B	57	530R, 530F, 1912R, 18S-CL-R2, 18S-CL-F2, 18S-CL-R5, 18S-CL-F8, ITS-CL-F2, rDNA15.8S, 5.8SF, AB28, V2R, 28S-CL-F1, D2A, 28S-CL-F3, 28S-CL-R3, 28S-CL-R1, and 28S-CL-F2
*Litylenchus* sp.	Live specimen 104H88, isolated from the leaf of beech, Perry, Ohio	18S-CL-F3 and 28S-CL-R	50	530R, 530F, 1912R, 18S-CL-R2, 18S-CL-F2, 18S-CL-R5, 18S-CL-R7, ITS-CL-F2, rDNA15.8S, V2R, 28S-CL-F3, 28S-CL-R1, 28S-CL-F2, 1006R and 28S-CL-R
*Meloidogyne incognita*	Live J2 specimen Me47 from the isolate, RKN Race 1, originally was collected in Maryland and maintained with ‘PA-136’ pepper in greenhouse pots	18S-CL-F3 and D3B	57	530R, 530F, 1912R, 18S-CL-R2, 18S-CL-F2, 18S-CL-F7, 18S-CL-R5, 18S-CL-R7, ITS-CL-F2, 5.8SF, V2R, 28S-CL-R1, 28S-CL-F2 and 1006R
*Pratylenchus scribneri*	Live specimen Pr1 from a culture maintained with corn root explant; originally collected from soil in Beltsville, Maryland	18S-CL-F3 and D3B	57	18S-CL-F3, 530R, 530F, 1912R, 18S-CL-R2, 18S-CL-F2, 18S-CL-F7, 18S-CL-R5, 18S-CL-R7, ITS-CL-F2, rDNA15.8S, 5.8SF, Ab28, V2R, D2Ar, D2A, 28S-CL-F3, 28S-CL-R3, 28S-CL-R1, 28S-CL-F2 and 28S-CL-R
*Radopholus similis*	Live specimen 31G1, obtained by USDA – APHIS – PPQ interception from *Anthurium* in Kurtistown, Hawaii	18S-CL-F3 and 28S-CL-R	50	530R, 530F,1912R, 18S-CL-R2, 18S-CL-F2, 18S-CL-F7, 18S-CL-R5, 18S-CL-R7, ITS-CL-F2, rDNA15.8S, 5.8SF, AB28, V2R, D2AR, 28S-CL-F1, 28S-CL-F3, 28S-CL-R1, 28S-CL-F2 and 1006R
*Xiphinema* sp.	Live specimen 06D2, isolated from soil in Clarksville, Maryland	18S-CL-F3 and D3B	50	18S-CL-F3,530R, 530F,1912R, 18S-CL-R2, 18S-CL-F2, 18S-CL-F7, 18S-CL-R5, 18S-CL-R6, ITS-CL-F2, 5.8S-CL-F1, 5.8S-CL-R1(XitsS3), V2R, D2AR,D2A, 28S-CL-F3, 28S-CL-R1, 28S-CL-F2, and 1006R
*Xiphinema* sp.	Live specimen 104F83, isolated from the rhizosphere of bamboo, Beltsville, Maryland	18S-CL-F3 and D3B	57	18S-CL-F3, 530R, 530F,1912R, 18S-CL-R2, 18S-CL-F2, 18S-CL-F7, 18S-CL-R5, 18S-CL-R6, ITS-CL-F2, 5.8SF, 5.8S-CL-R1(Xist3), V2R, D2AR, 28S-CL-F2, 1006R and 28S-CL-R

### PCR amplification and DNA Sequencing

**Figure 1:**
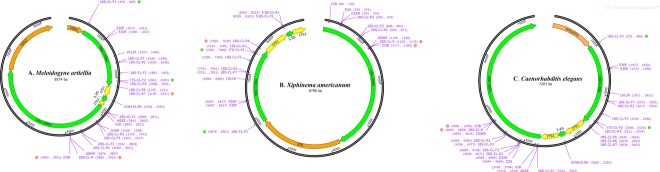
Position diagram of primers. (A): rDNA (AF248477), *Meloidogyne artiellia*; (B): rDNA (AY580056), *Xiphinema americanum*. (C): rDNA (X03680), *Caenorhabditis elegans*. Created with SnapGene software (GSL Biotech, Chicago, IL; available at snapgene.com) and assembled with Adobe Photoshop CC 2018 (Adobe Systems Incorporated, San Jose, CA).

Each PCR reaction was prepared with 2 µl of DNA extract and 23 µl of the PCR master mix [H_2_O: 16.375 µl; 10x DreamTaqTM (Thermo Fisher Scientific, Waltham, MA, USA) buffer: 2.5 µl; dNTP mix, 2.0 mM each: 2.5 µl; 10 µM forward primer: 0.75 µl; 10 µM reverse primer: 0.75 µl; 0.625U DreamTaqTM Hot Start DNA Polymerase 5 U/µl: 0.125 µl, assembled per manufacturer’s manual] containing the primer pairs 18S-CL-F3 and either D3B or 28S-CL-R (Tables 1 and 2). PCR was carried out within a Bio-Rad MJ Mini or C1000 Touch gradient thermal cycler (Bio-Rad Laboratories, Hercules, CA). The PCR conditions were 95°C for 3 min; 36 cycles of 95°C for 30 sec, annealing temperature (T_a_) at 57°C or 50°C (Table 1) for 45 sec, and 72°C for 3 min; and final extension at 72°C for 7 min. PCR products were visualized with the Lonza FlashGel^TM^ DNA system (VWR International, Radnor, PA) and then treated with ExoSAP-IT reagent (Affymetrix, Inc, Santa Clara, CA) according to manufacturers’ protocols. Direct DNA sequencing was performed bidirectionally with multiple primers (Tables 1, 2 and Fig. 1) and an ABI BigDye® Terminator v3.1 kit and in an ABI 3730xl DNA Analyzer (Applied Biosystems, Foster City, CA, USA) owned by the USDA Systematic Entomology Lab, Beltsville, MD. The ribosomal primers, 18S-CL-F7, 18S-CL-R6, 18S-CL-R7, 18S-CL-F8, 5.8S-CL-F1, 5.8S-CL-R1, 28S-CL-F1, 28S-CL-R3, 28S-CL-F3, 28S-CL-R1, 28S-CL-F2 (Table 2) were newly designed with Geneious ver. 10.1.1 (BioMatters, Auckland, New Zealand).

**Table 2.** Ribosomal primers used for PCR and sequencing.

Primers	Direction	Loci	Sequence (5′-3′)	PCR	Sequencing	References
18S-CL-F3	F	18S	CTTGTCTCAAAGATTAAGCCATGCAT	✓	✓	Carta and Li (2018)
D3B	R	28S	TCGGAAGGAACCAGCTACTA	✓	✓	Nunn (1992)
28S-CL-R	R	28S	CAGCTACTAGATGGTTCGATTAGTC	✓	✓	This study
18S-530F (530F)	F	18S	AAGTGTGGTGCCAGCAGCCGC		✓	Reverse complement of 530R
18S-530R (530R)	R	18S	GCGGCTGCTGGCACCACACTT		✓	Thomas et al. (2011)
1912R	R	18S	TTTACGGTCAGAACTAGGG		✓	Holterman et al. (2006)
18S-CL-R2	R	18S	GTTGAGTCAAATTAAGCCGCA		✓	Carta and Li (2018)
18S-CL-F7	F	18S	TGCGGCTTAATTTGACTCAAC		✓	This study
18S-CL-F2	F	18S	CTGTGATGCCCTTAGATGTCC		✓	Carta and Li (2018)
18S-CL-R5	R	18S	GCGGTGTGTACAAAGGGCAGGGAC		✓	Carta and Li (2018)
18S-CL-R6	R	18S	ACCTTGTTACGACTTTTACTTCCTCTA	✓	✓	This study
18S-CL-R7	R	18S	ACCTTGTTACGACTTTTGCCCGGTTCA	✓	✓	This study
18S-CL-F8	F	18S	TGAACCGGGCAAAAGTCGTAACAAGGT		✓	This study
ITS-CL-F2	F	ITS	ATTACGTCCCTGCCCTTTGTA	✓	✓	Carta and Li (2018)
5.8S-CL-F1	F	5.8S	GATTCCATCATTCTAAGC		✓	This study
5.8S-CL-R1	R	5.8S	ACCGCTTAGAATGATGGAATC		✓	This study
rDNA15.8S	F	5.8S	ACGAGCCGAGTGATCCACCG		✓	Cherry et al. (1997)
5.8SF	F	5.8S	CGGTGGATCACTCGGCTCGT		✓	Reverse complement of rDNA1.58S
AB28	R	28S	ATATGCTTAAGTTCAGCGGGT		✓	Joyce et al. (1994)
28S-CL-F1	F	28S	CTGAACTTAAGCATATCAGTAAGC		✓	This study
VRAIN 2R (V2R)	R	28S	TTTCACTCGCCGTTACTAAGGGAATC		✓	Vrain et al. (1992)
D2AR	R	28S	ACTTTCCCTCACGGTACTTGT		✓	Reverse complement of D2A
D2A	F	28S	ACAAGTACCGTGAGGGAAAGT		✓	Nunn (1992)
28S-CL-R3	R	28S	GCAACTTTCCCTCACGGTACTTG			This study
28S-CL-F3	F	28S	AAGAGAGAGTTAAAGAGGACGTGAA		✓	This study
28S-CL-R1	R	28S	ACTCCTTGGTCCGTGTTTCAAG		✓	This study
28S-CL-F2	F	28S	CGACCCGTCTTGAAACAC		✓	This study
28S-1006rev (1006R)	R	28S	GTTCGATTAGTCTTTCGCCCCT		✓	Holterman et al. (2008)

## Results

**Figure 2:**
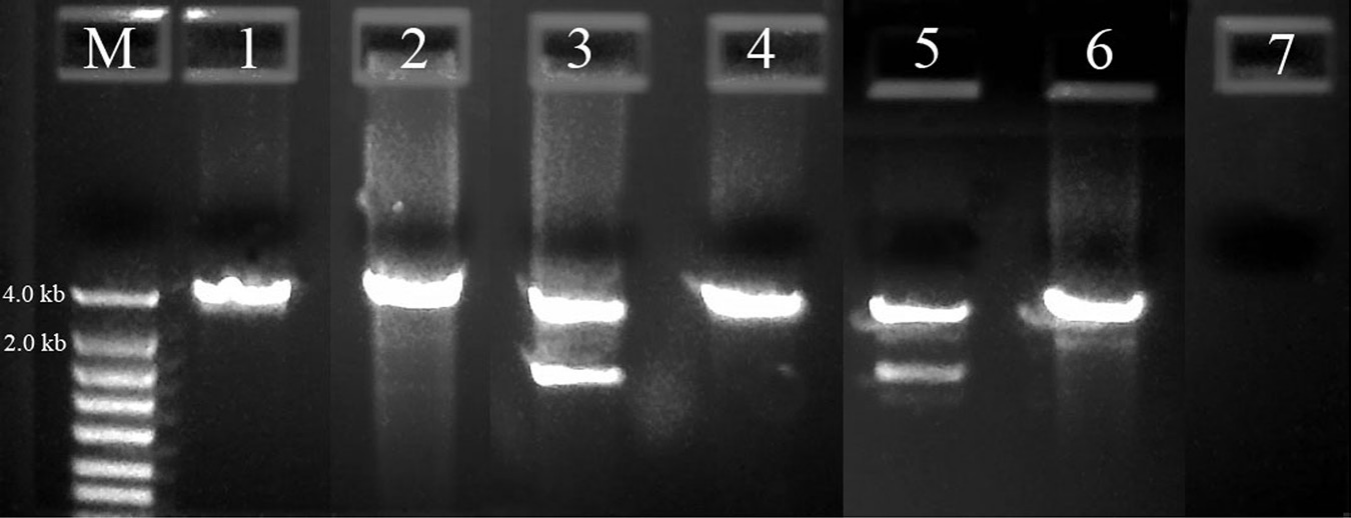
PCR Amplification of 3.3 to 4.2 kb of rDNA from agriculturally important nematodes. M: DNA markers; 1: *Heterodera orientalis* 104F80; 2: *Xiphinema* sp. 104F83; 3: *Hoplolaimus* sp. 104G35; 4: *Helicotylenchus* sp. 104G36; 5: *Meloidogyne incognita* Me47; 6: *Pratylenchus scribneri* Pr1 and 7: Negative control.

The forward PCR primer for this long rDNA target was 18S-CL-F3, the reverse primer was D3B and a newly designed 28S primer, 28S-CL-R, was applied as well (Table 2). Figure 2 shows that this long rDNA target was amplified by PCR in six taxa from agriculturally important nematodes. The internal sequencing primers with reading overlaps (Fig. 1) were tested by using cycling amplification. The rDNA sequences resulted from *Helicotylenchus* sp. 104G36 (MK292128), *Heterodera orientalis* 104F80 (MK292130), *Hoplolaimus* sp. 104G35 (MK292131), *Meloidogyne incognita* Me47 (MK292132), *Pratylenchus scribneri* Pr1(MK292133), and *Xiphinema* sp. 104F83 (MK292136) with high coverage in each base were deposited to GenBank. Additionally, another 11 agriculturally important nematodes were also tested by using the same approach described above when they became available, and the resulting sequences with Accession No. MK292123 for *Aphelenchoides fragariae* 104J12, MK292121 for *Bursaphelenchus* sp.104J7, MK292122 for *Bursaphelenchus xylophilus*104H46, MK292124 for *Crossonema* sp.104G38, MK292125 for *Ditylenchus dipsaci* 85A5, MK292126 for *Ditylenchus* sp. 85C1, MK292127 for *Ecumenicus* sp.85G11, MK292129 for *Heterodera glycines* H20, MK292138 for *Litylenchus* sp. 104H88, MK292134 for *Radopholus similis* 31G1, and MK292135 for *Xiphinema* sp. 06D2 were deposited to GenBank as well.

## Discussion

An approximate base-pair match between primer and template DNA is required for Taq DNA polymerase to begin an efficient PCR amplification cycle (Watson, 1971; Kwok et al., 1994; Wu et al., 2009; Wright et al., 2014). The taxonomic universality of 18S-CL-F3, the forward primer selected for the single primer pair approach, has been presented previously from taxa in Tylenchida and Dorylaimida (Carta and Li, 2018). We have not seen any failures of PCR amplification from the taxa tested with 18S-CL-F3 since it was designed in our lab. The D3B segment primer was selected because it is a universal reverse primer for amplifying the D2D3 segment of 28S across Nematoda (Nunn, 1992). Primer 28S-CL-R was also designed as a substitute for D3B. The primer pair 18S-CL-F3 and either D3B or 28S-CL-R is the primary key to the success of the single primer pair approach achieved in this study. To our knowledge, this is the first report of using a single primer pair to amplify this long rDNA target in diverse agriculturally important nematodes.

Although this long rDNA target was amplified successfully with a traditional Taq DNA polymerase used in the single primer pair approach, it should be noted that Taq DNA polymerase lacks 3′-5′ exonuclease activity and is incapable of proofreading any misincorporated nucleotides during PCR (Tindall and Kunkel, 1988). This inability could make the Taq dissociate from its template DNA before the extension is completed and subsequently limits the size of the amplicon (Arezi et al., 2003). Therefore, it is desirable that thermal proofreading DNA polymerase, Pfu, Vent or others, along with a Taq or a blend of both enzymes be employed in the single primer pair approach when the PCR amplification of the long target becomes difficult (Barnes, 1994; Cheng et al., 1995). Strong non-specific amplicon bands in the specimens 104G35 and Me47 (Lane 3 and Lane 5 in Figure 2, respectively) were observed, however, their sequence reads were not interrupted by the non-target amplicons (data not shown). This is because the ribosomal internal sequencing primers applied in this study can only recognize the rDNA amplicon that has these primer binding sites. Therefore, choosing the internal sequencing primers is critical for the direct DNA sequencing in the single primer pair approach. Primer 530R (Table 2) was selected and 28S-CL-F2 designed specifically to read the 5′-end and 3′-end of this 3.3 to 4.2 kb rDNA amplicon, respectively, during cycling with BigDye® reagents. These two primers were particularly useful for sequencing because the PCR primers may not be used as sequencing primers when non-specific bands (amplicons) occur. Both 18S-CL-R7 and 18S-CL-R6 were designed as sequencing primers initially for Tylenchida and Dorylaimida, respectively. Moreover, they could also be paired with 18S-CL-F3 to amplify the 18S in near-full length as needed; and 28S-CL-R could be paired with ITS-CL-F2 for either the amplification of ITS, or for 28S (D1D2D3) or both to meet different goals.

In this study, we demonstrated the PCR amplification of this 3.3 to 4.2 kb rDNA in 17 agriculturally important nematodes using the single primer pair approach. The taxonomic coverage by these two single primer pairs revealed in this study suggests that they may also be valid for other plant-parasitic nematode taxa in Tylenchida and Dorylaimida. Additionally, this ability was seen in several taxa in Rhabditida with the 18S-CL-F3 and 28S-CL-R pair (data not shown). This study also provides the internal ribosomal sequencing primers that are well positioned in the target rDNA (Table 2 and Fig. 1) with high base coverages to acquire the high quality rDNA sequences spanning from 18S in near full length, ITS1 in full length, 5.8S in full length, ITS2 in full length, to the D1, D2, and D3 segments of 28S at once, which would facilitate deep phylogenetic analysis and accurate taxonomic identification by using individual specimens. Particularly, the D1 segment between ITS2 and 28S and the flanking sequence between 18S and ITS1 were fully revealed by the single primer pair approach, while they are a blind spot in the multiple primer pair approach. These ribosomal primer pairs could also be utilized for meta-barcoding by targeting this long rDNA target from environmental nematode DNA samples. The resulting amplicons could be sequenced using different Next Generation Sequencing platforms such as PacBio (Pacific BioSciences, Menlo Park, CA) and Nanopore (Oxford Nanopore Technologies, Oxford, UK) for long reads.
